# Molecular Association Between Short Linear Maltodextrin and Ferulic Acid and the Exploration of Its Applicability

**DOI:** 10.3390/polym18020166

**Published:** 2026-01-07

**Authors:** Shigesaburo Ogawa, Daisuke Sugitani, Minenosuke Matsutani, Mizuho Takayashiki, Atsushi Kawano

**Affiliations:** 1 Department of Food, Aroma and Cosmetic Chemistry, Faculty of Bio-industry, Tokyo University of Agriculture, 196, Yasaka 099-2493, Hokkaido, Japan; 2 Research & Engineering Department, Showa Sangyo Co., Ltd., 2-20-2 Hinode, Funabashi-shi 273-0015, Chiba, Japan

**Keywords:** linear maltodextrin, ferulic acid, molecular association, radical scavenging, UV-protection

## Abstract

Short linear maltodextrin (SLMD) mixtures, which are modified from starch, comprise approximately 10 linear glucose molecules. In this study, we explored the noncovalent molecular association of SLMD with ferulic acid (FA) in aqueous and solid systems, as well as its applicability to water-in-oil (W/O) emulsion systems. Results showed that SLMD interacts with FA at a 1:1 molar ratio with an average equilibrium constant of 13.3 M^−1^ in pure water. Changes in ellipticity in the involved circular dichroism absorption spectrum and nuclear magnetic resonance spectroscopy revealed that multipoint direct interactions exist between SLMD and FA suggesting complex formation through inclusion. Complexation does not impede the radical scavenging ability of FA; instead, there is an additive effect with a slight contribution from SLMD. SLMD crystals with a high FA content were obtained for B-type amylose. However, no strong interaction between the solid forms of SLMD and FA was recognized. For both SLMD aq. and W/O emulsions with different FA concentrations, the UV protection effect increased due to the solubility enhancement of FA by SLMD. Overall, this study demonstrates the ability and potential importance of SLMD to associate with functional components in water and solid systems and the applicability to emulsified systems.

## 1. Introduction

Starch is a mixture of polysaccharides comprising amylopectin and amylose [1]. Although both structures comprise glucose, the former has a branched structure via an α-1,6-glucosidic linkage and the latter has a linear structure via an α-1,4-glucosidic linkage. The differences in the proportions of these structures directly affect the fundamental crystal structures as well as the resultant basic properties such as digestion, gelling, and swelling of starch [2,3]. This has led to extensive research on the characteristics of starch resources and an improved understanding of their structural components. Furthermore, amylose forms a helical structure and complex with other components by incorporating them into its structure [4]. The importance of its impact on processing and functionality has prompted active research; however, the structural clarification of this complexation still remains challenging [5,6,7,8,9].

**Figure 1:**
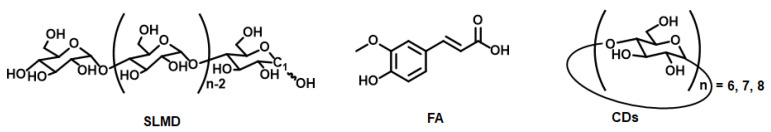
Chemical structures of (**left**) short linear maltodextrin (SLMD), (**middle**) ferulic acid (FA), and (**right**) cyclodextrins (CDs), respectively.

In addition, recent advancements in biotechnology have enabled enzymatic starch hydrolysis to produce various starch-derived hydrolysis products, some of which exhibit high potential for application in cosmetics and pharmaceuticals as well as foods [10,11,12,13]. In recent years, amylose preparative methods with controlled residue glucose units and structure have been developed on an industrial scale, and a resultant product is now commercialized. For instance, Kawano et al. successfully prepared a product comprising short linear maltodextrin (SLMD, Figure 1(left)) with an average polymerization degree (n) of 8.49 and a narrow molecular weight distribution through a stepwise enzymatic reaction [14]. SLMD exhibited complexation capacity with iodine, suggesting the formation of the helical structures as formed by high-molecular-weight amylose (high-amylose). Furthermore, high-amylose exhibits polymorphism, and A-, B-, and V-type patterns are well documented [15]. Both A- and B-type patterns comprise the double-helix structure of amylose, whereas the A-type pattern is packed with monoclinic unit cells, and the B-type pattern is packed hexagonal with large pores in the center area. The V-type pattern is packed with the single helix structure of amylose. Interestingly, SLMD also exhibits polymorphism such as A- and B-type patterns [16]. Nevertheless, the molecular association behavior of SLMD with functional components other than iodine in both solution and solid state remains unclear.

The interaction between starch and polyphenols has received considerable attention due to its practical importance, with several studies proposing computational simulations of their interaction mechanisms [7,17,18]. Ferulic acid (FA, Figure 1 (middle)) is a component that has been extensively investigated for mixture with starch [6,17,18,19,20,21]. Remarkably, Hao et al. proposed the interaction mechanism between high-amylose in corn starch and FA using computer simulations [17]. Nevertheless, there are extremely limited experimental studies that have directly investigated the molecular interaction between amylose structures and polyphenols, including FA. This may be due to the challenges in sample preparation of high-purity amylose with a narrow molecular weight distribution. Conversely, cyclic oligosaccharides such as cyclodextrin (CD, Figure 1 (right)), which exhibit high solubility in water and the ability to form complexes with FA, have been extensively investigated, and the molecular association behaviors have been clearly demonstrated [22,23,24].

In this study, we hypothesized that using SLMD could provide experimental information on the nature of the interactions between amylose and FA. This information is anticipated to contribute to the development of starch science and advance the use of starch hydrolysis compounds in fields such as cosmetics and pharmaceuticals. On the basis of this viewpoint, we explored the presence or absence of interactions between SLMD and FA in both aqueous and solid systems, inferred the inclusion formation state, and investigated the effects on the antioxidant activity of FA. Furthermore, since FA is known to function as a UV-absorbing agent, we also examined the UV protection effects of SLMD–FA mixed systems. In addition to the aqueous system, we evaluated its usefulness in water-in-oil (W/O) emulsion systems.

## 2. Materials and Methods

**Figure 2:**
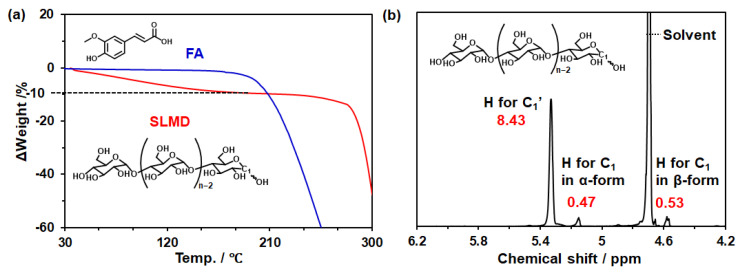
Characterizations of commercially available SLMD and FA. (**a**) Thermogravimetric (TG) analysis of commercial SLMD (6.3 mg) and FA (4.1 mg). (**b**) ^1^H-nuclear magnetic resonance (NMR) spectrum of commercially available SLMD (D_2_O, 25 °C).

**Chemicals.** Powdered SLMD sample (AmyloSoln) manufactured using a previously described method [14] with slight modification was supplied by Showa Sangyo Co., Ltd. (Tokyo, Japan) and used without further purification. Based on the results of Figure 2, the SLMD purity was calculated to be 90 wt%, the average polymerization degree was approximately 9.7, and the average molecular weight was approximately 1547. FA, a type of polyphenol and antioxidant component, was purchased from Tokyo Kasei Co., Ltd. (Tokyo, Japan). In addition, CD was employed for comparison in some experiments. α-Cyclodextrin (α-CD) and γ-cyclodextrin (γ-CD) were purchased from Tokyo Chemical Industry Co., Ltd. (Tokyo, Japan), and β-cyclodextrin (β-CD) was purchased from Fujifilm Wako Pure Chemical Industries, Ltd. (Osaka, Japan) and used as supplied. The emulsifier, NIKKOL HEXAGLYN PR15 ((poly)glycerol Fatty Acid Ester type emulsifier), was used as supplied by Nikko Chemicals Co., Ltd. (Tokyo, Japan). Hexadecane (TCI, Tokyo, Japan) or triethylhexanoin (TGO, Kao Corp., Tokyo, Japan) was used as the oil phase solvent. For nuclear magnetic resonance (NMR) spectroscopy measurements, heavy water (D_2_O) purchased from Tokyo Chemical Industry Co., Ltd. (Tokyo, Japan), was used, and distilled water (H_2_O) purchased from Fujifilm Wako Pure Chemical Industries, Ltd. (Osaka, Japan), was used in other experiments. In the ABTS radical scavenging assay, 2,2′-azino-bis (3-ethylbenzothiazoline-6-sulfonic acid) and potassium peroxodisulfate purchased from Fujifilm Wako Pure Chemical Industries, Ltd. (Osaka, Japan), were used. Other organic solvents used in this study were of analytical grade. **Solution preparation.** To prepare the SLMD–FA complex, an excess amount of FA was added to an aqueous solution of SLMD with different SLMD concentrations. H_2_O or D_2_O were used as water sources depending on the experimental object. After 16 h of stirring at ambient atmosphere, the sample mixtures were filtered through a 0.45 μm nanopore membrane filter (DISMIC^®^-13HP, Toyo Roshi Kaisha, Ltd., Tokyo, Japan) to yield SLMD–saturated FA aq. CD–saturated FA complexes were prepared in a similar manner, with the concentrations ranging from 0.1 to 5 mM for α-CD and β-CD, and from 0.1 to 2.5 mM for γ-CD. **Solid preparation.** Three preparation protocols (Methods A, B, and V) were conducted to examine whether SLMD–FA molecular complexes formed in the solid state [16,25,26]. **Method A:** First, 10 wt% of SLMD aq. (50 mL) was heated in a water bath at 80 °C for 30 min, and FA (0 or 124.2 mg) was added and dissolved. Then, the solution was cooled to 20 °C and acetone or isopropyl alcohol (IPA) was gradually added, during which white crystals precipitated [25]. **Method B:** 30 wt% of SLMD was prepared by adding SLMD into distilled water followed by ultrasonication at 20 °C without prior heating. FA (0 or 199.3 mg) was then added into the solution and dissolved by magnetic stirring. After storing at 4 °C for 24 h, a white precipitate was obtained [16]. **Method V:** To 95% DMSO aq. (100 mL) at 90 °C, 5 g SLMD was added and dissolved. The hot solution was poured into FA (0 or 1.26 g) containing EtOH or IPA sol. (250 mL) and subjected to vigorous magnetic stirring [26]. Each precipitate resulting from Methods A, B, and V was obtained by membrane filtration with a 0.45-μm-pore filter. Crystals obtained via Method B were washed thrice with acetone, EtOH, or IPA. **W/O emulsion preparation.** SLMD-FA aq. was prepared with various SLMD and FA concentrations (2 g) and emulsifiers (0.25 g) in hexadecane, as well as in saturated FA-containing or FA-non-containing TGO (3 g), with or without curcumin. These mixtures were vortex-mixed for 1 min. Ultrasonic homogenization was carried out for 8 min at 20 °C using a UH-50 (50 W, 20 kHz, SMT Co., Ltd., Tokyo, Japan) and an MS-3 sonicator microtip for the UV protection test. **Thermogravimetric (TG) analysis.** Because the occurrence of water hydration results in calculation errors of sample concentrations, TG analysis was performed at a rate of 10 °C/min in an air atmosphere using TG8122 (Rigaku Corp., Tokyo, Japan) to confirm the amount of solvent in commercial SLMD and FA. Each sample was measured in duplicate. **Solubility test.** FA concentration was determined by absorption at 332 nm using Thermo Scientific^TM^ Varioskan^TM^ LUX (Vantaa, Finland) after adding DMSO to the filtrate to prepare 90% DMSO SLMD aq. The calibration curve of FA in 90% DMSO aq. was used for determining the FA concentration of the solution. Average values were obtained from three experiments, and the standard deviation was calculated using Excel. A similar method was used for the solubility test of FA in CD aq. **Circular dichroism absorption spectroscopy.** Circular dichroism absorption spectroscopy was performed at 20 °C and at a rate of 200 nm/min using a 1 cm cell and J-820 (JASCO Co., Tokyo, Japan) on SLMD-FA aq. between 240 and 400 nm. Because saturated FA solution was too high to detect accurate value, 63 mM SLMD–saturated FA aq. or saturated FA aq. were diluted about 20 times with 63 mM SLMD aq. or distilled water. Then, the diluted solution was measured, wherein a high FA concentration indicated measurement error in ellipticity. Baseline correction was performed using pure water. **^1^H-NMR measurement.** H-NMR spectroscopy was performed at various temperatures using an ECA 600 (JASCO Corp., Tokyo, Japan) with 16 scans to determine the average molecular weight of SLMD and also to investigate the concentration-dependent behavior of SLMD–FA aq. **ROESY NMR.** Two-dimensional (2D) ROESY NMR experiments were conducted on 63 mM SLMD–saturated FA aq. solution, FA aq. solution, and 63 mM SLMD aq. solution at 20 °C. The experiments had a mixing time of 250 ms and a relaxation time of 1.5 s, and 4 scans were performed. Each sample was prepared as described in the solution preparation section. The FA concentration was measured spectroscopically after adding DMSO to 90% DMSO aq. solution. **ABTS radical scavenging test.** The ABTS biradical was used for the radical scavenging test [27,28]. Briefly, ABTS (117.8 mg) was dissolved in water (30.6 mL), and potassium peroxodisulfate (28.2 mg) was added to activate the ABTS radical. The solution was then stored overnight in the dark, after which the ABTS radical solution was diluted to the appropriate concentration for spectroscopic measurement. The ABTS radical scavenging test was performed by adding SLMD–FA aq. or SLMD aq. with different SLMD concentrations to ABTS aq. UV-vis spectra and absorptions were measured for each solution at 732 nm after 2 h. The experiment was conducted three times, and the average values are plotted. **Differential scanning calorimetry (DSC).** The endothermic enthalpy (Δ*H*) and phase transition temperature were determined using DSC 8230 (Rigaku Corp., Tokyo, Japan) at a rate of 5 °C/min in an air atmosphere. Each sample was measured in duplicate. **Fluorescent microscopy observation**. For W/O emulsion samples, fluorescent microscopic images were captured using a BZ-X800 analyzer (Keyence Corp., Osaka, Japan) under GFP light excitation (488 nm). **UV protection effect**: 0.4 mL of SLMD-FA aqueous or SLMD-FA aqueous–oil mixtures were poured onto SPL cell culture dishes (3.5 cm diameter; Nippon Genetics Co. Ltd., Tokyo, Japan) and placed on the detector of a TM-213 ultraviolet intensity meter (MK Scientific, Inc., Kanagawa, Japan). The sample was irradiated with UV-B light (310 nm) to measure the UV protection effect in terms of transmittance percentage. In this case, 0% protection indicates that the detector was exposed approximately 0.34 mW of UV-B light. **Emulsion stability test**: The stability of the W/O emulsion was evaluated by observing how the oil separated. Stability was assessed based on the thickness of the oil phase when observed on its own. A smaller thickness indicates higher stability. **Partition concentration in the water and oil phases**: The partition concentration of FA in TGO was studied against SLMD-aq. in the absence of emulsifiers. 0.75 mL of saturated SLMD-FA aqueous solution and TGO without FA was mixed with a vortex for 10 min, after which centrifugation was applied. The resulting phases were mixed with DMSO and the FA concentration was determined by measuring the absorption at 330 nm using a Thermo Scientific™ Varioskan™ LUX (Vantaa, Finland) after adding DMSO to dilute the FA concentration. The partition concentration (P) of TGO phase was calculated as [FA in oil]/[FA in SLMD aq.]. **Statistical analysis**: A paired Student’s t-test was performed on the paired plots, and differences were considered statistically significant at *p* < 0.05 or *p* < 0.005. Additionally, regression analysis was performed using concentration or the log of concentration as the independent variable, respectively.

## 3. Results and Discussion

### 3.1. Confirmation of the State of Commercial Samples

As carbohydrates and polyphenols exhibit various hydration states, it is essential to first determine the hydration state of the sample to discuss their concentrations in aqueous solutions accurately. Hence, we first performed TG analysis on SLMD and FA to determine their moisture content. Using the TG profiles depicted in Figure 2a, the moisture content of SLMD was calculated as 10%, indicating that the commercially available sample had a purity of 90% of SLMD. Conversely, FA was found to be in an anhydrous state (Figure 2a).

Next, we determined the average molecular weight of SLMD using ^1^H-NMR measurement. Using the ^1^H-NMR spectrum shown in Figure 2b, the number of hydrogen atoms on the C1 carbon of terminal glucose in SLMD can be calculated as the sum of the α-form and β-form structures [29], and the average polymerization degree can be determined from the ratio of this number to the total number of hydrogen atoms on the C1 carbon of other glucose molecules. Results showed that the average polymerization degree was *ca.* 9.7 and that the average molecular weight was *ca*. 1547. This value is comparable to the previously reported value of average n = 8.49 estimated by gel permeation chromatography measurement [14] and is considered a reasonable result. Therefore, this average molecular weight of 1547 was subsequently used for determining the aqueous solution concentration of SLMD.

### 3.2. Confirmation of Complexation from Solubility Test and Circular Dichroism

**Figure 3:**
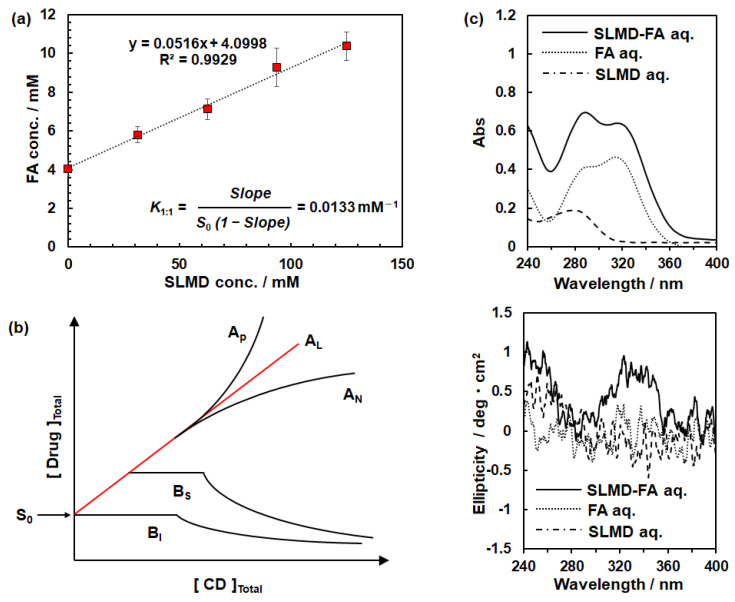
Confirmation of complexation behavior. (**a**) Effect of SLMD on the solubility of FA in water. (**b**) Representative drug solubility–CD concentration relation which was reproduced from [30], MDPI, 2018. (**c**) (above) Absorption spectrum and (below) ellipticity for circular dichroism absorption measurement.

The concentrations of saturated FA for each solution relative to SLMD aq. at concentrations of 125 (18 wt%), 94, 63, 31, and 0 mM were estimated (Figure 3a). The FA concentration increased linearly with increasing SLMD concentration. A high linearity coefficient of >0.99 confirmed a strong linear correlation between SLMD concentration and FA concentration.

In general, the interaction between CD, a cyclic oligosaccharide that forms inclusion complexes by incorporating other components, and guest (e.g., drug) is classified as illustrated in Figure 3b [30]. The A_L_ type corresponds to the type where CD and the drug interact in a 1:1 ratio, and the relation between SLMD and FA in this study was distinctly classified as the A_L_ type. The average equilibrium constant (*K*_1:1_) was calculated as 13.3 M^−1^ using the equation shown in Figure 3a, where Slope is the slope of the straight line representing the relation between SLMD and FA concentration and S_0_. The *K*_1:1_ values for the interaction between CDs and FA were 397, 103, and 765 M^−1^ for α-CD, β-CD and γ-CD, respectively.

Furthermore, when the guest is encapsulated by CD, it is reported that the guest absorption transition becomes chirally perturbed in the proximity of a CD molecule and an induced circular dichroism signal appears in the absorption wavelength region of the guest molecule [31,32]. Therefore, we measured the circular dichroism absorption spectra for SLMD and FA solutions as shown in Figure 3c, where the upper panel depicts the absorption spectrum, and the lower panel depicts the wavelength dependence of the ellipticity. The ellipticity clearly changes for the SLMD-FA mixture at wavelengths of ≤360 nm, where FA absorption is detected, whereas no clear change was recognized for SLMD aq. and FA aq. solutions. This suggests that FA undergoes a clear twisting of its overall structure due to the impact of the chirality of SLMD. Since FA is a non-chiral compound, the appearance of the peak should be attributed to induced circular dichroism through interacting with SLMD.

### 3.3. Confirmation of Complexation from Nuclear Magnetic Resonance Spectroscopy

Recently, in terms of molecular association between amylose and FA, the DFT calculation proposed a model where FA is encapsulated within the helical structure of amylose and exerts multipoint interactions [17]. Nevertheless, there has been no experimental evidence on aqueous solutions. Typically, NMR spectroscopy has also been used to explore the interactions between CD and FA [22,23,24]; hence, NMR analysis was performed in the present study to investigate the interactions between SLMD and FA.

**Figure 4:**
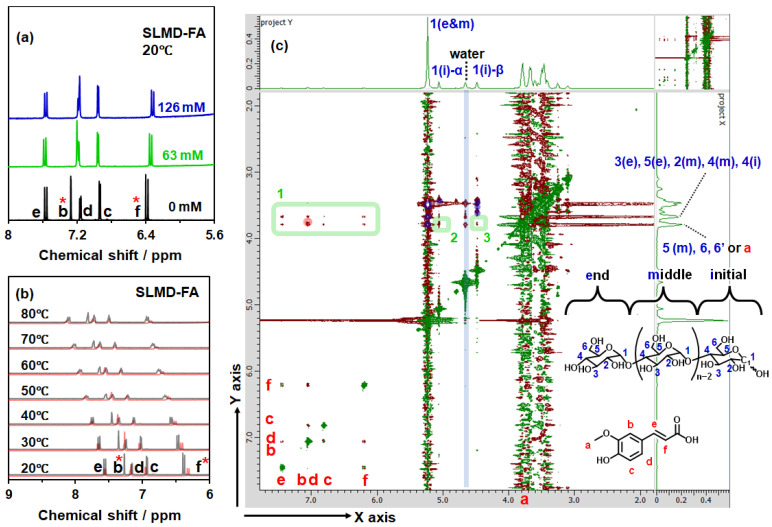
^1^H-nuclear magnetic resonance (NMR) spectra of (**a**) SLMD-saturated FA aq. at different SLMD concentrations at 20 °C and (**b**) ^1^H-NMR spectra of temperature dependence (D_2_O) and (**c**) ROESY two-dimensional (2D) NMR analysis of 63 mM SLMD–5.9 mM FA aq. at 20 °C (D_2_O). Proton signal of H_2_O at 4.69 ppm was used as a standard of each measurement. The asterisk (*) indicates that the signal exhibits concentration dependence in Figure 4a. Each proton was assigned alphabetically with red or numerically with blue as shown in Figure 4c. The red and blue circles in Figure 4c were observed for saturated FA aq. (20 °C, D_2_O) and 63 mM SLMD aq. (20 °C, D_2_O), and except for these, the area surrounded by green was newly observed evidence of the interaction between SLMD and FA for 63 mM SLMD–5.9 mM FA aq. (20 °C, D_2_O).

Figure 4a depicts the results of ^1^H-NMR measurements at 20 °C in the presence of saturated FA at SLMD concentrations of 0, 63, and 126 mM. Each proton is assigned alphabetically or numerically as illustrated in Figure 4c. In the absence of SLMD, b and d are clearly recognized as distinct individual signals. However, as the SLMD concentration increases, the chemical shift of b decreases and approaches that of d and further changes to a value smaller than that of d. Moreover, a concentration-dependent decrease in chemical shift was confirmed for f. Other signals, c, d, and e, appeared to shift to slightly higher positions; however, no apparent changes were detected between concentrations of 63 and 126 mM, and no clear concentration dependence was confirmed. Hence, it was considered that at least b and f are considerably affected by the interaction between SLMD and FA at 63 mM SLMD concentration. This trend was consistent across various temperatures ranging from 20 °C to 80 °C, suggesting that SLMD and FA interact similarly even at 80 °C (Figure 4b). This suggested that the FA–starch interaction cannot be ignored even under heating conditions. As it was unclear which protons directly contribute to the interaction based solely on the concentration dependence and temperature dependence depicted in Figure 3a,b, we conducted ROESY NMR measurements at 20 °C. However, no satisfactory information regarding the molecular association between SLMD and FA was obtained at 20 °C using NOESY NMR measurements or at 80 °C using ROESY NMR measurements.

In the 2D profile depicted in Figure 4c, signals indicating interactions between d and f and between b and e, for example, were observed in the chemical shift regions where both the X and Y axes exceed 6 ppm. Moreover, numerous signal plots indicating interactions between protons on SLMD were confirmed in the chemical shift regions where both the X and Y axes are ≤4.0 ppm. Conversely, the green region located in the area where the X-axis is ≥4.5 ppm and the Y-axis is between 3.6 and 3.9 ppm is probably an area indicating interactions between SLMD and FA. Except for the area enclosed by the red circle in the region where the X-axis is ≥6.2 ppm, the presence of direct interactions is obvious. The proton a on FA may exhibit a signal at 3.9 ppm; however, the signal spots in the green areas 2 and 3 strongly suggest a correlation with the proton signals of SLMD. In addition to f, where the shift was clearly confirmed in Figure 4a, the signals in c, e, and b or d were generally confirmed within the green area, indicating that they correlated with various protons in SLMD. This result suggests that SLMD forms a complex with FA through inclusion rather than interacting with specific regions of FA. In the complex formation of CD and FA through inclusion reported previously [22,23,24], all protons on FA are influenced by interactions with CD (Figure S1, Supplementary Materials). Although completely different chemical shifts are observed between γ-CD and FA, similar changes to those observed after SLMD addition are observed with α-CD and β-CD, suggesting that SLMD exhibits complexation behavior similar to that of α-CD or β-CD rather than γ-CD. It is most similar to β-CD, which likely correlates with the fact that the *K*_1:1_ of β-CD is closest to that of SLMD. However, SLMD’s inability to form a cyclic structure with covalent interactions results in a soft cavity and weak interaction with FA, resulting in a much lower *K*_1:1_.

Hao et al. reported DFT calculations showing that intermolecular interactions between the amylose structure and FA were mainly due to hydrophobic forces, hydrogen bonding, and van der Waals forces. In this study, the peak shifts of b and f may result from the interaction between the amylose structure and the phenolic hydroxy or carboxylate group, respectively. For the phenolic hydroxy group, the development of intermolecular interactions using the hydroxy group affects the methoxy group, which may have evolved an intramolecular interaction. The positional change after intermolecular interaction affects the proton b situation. Thus, the results and inferences obtained herein are consistent with the DFT calculation results of the amylose–FA complex reported by Hao et al. [17], strongly supporting the validity of their results that showed an inclusion state.

### 3.4. ABTS Radical Scavenging

**Figure 5:**
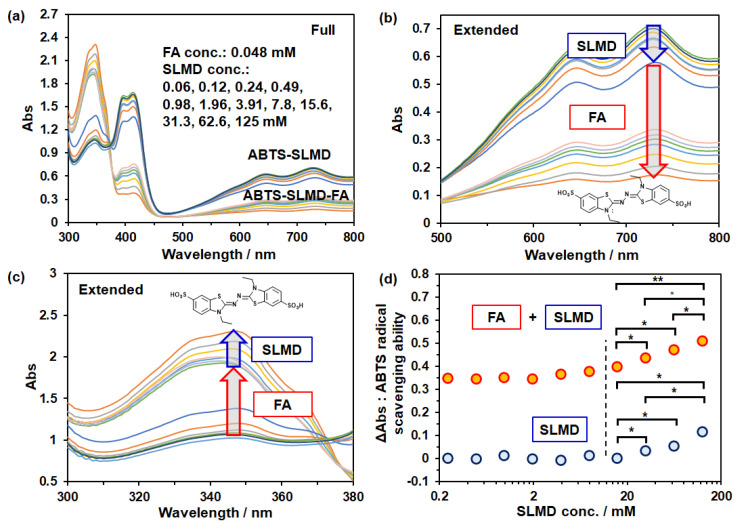
ABTS radical scavenging ability test. UV-vis absorption spectra after 2 h of reaction in the wavelength range of (**a**) 300–800 nm, (**b**) 500–800 nm, and (**c**) 300–380 nm and (**d**) the relation of ΔAbs vs. SLMD concentration. One asterisk (*) indicates *p*-value smaller than 0.05 (*p* < 0.05). Two asterisks (**) indicate *p*-value smaller than 0.005 (*p* < 0.005).

We next explored the impact of SLMD addition on the radical scavenging activity of FA. Although FA is a type of polyphenol that exhibits antioxidant activity, inclusion by CD can influence its antioxidant activity [28], indicating the importance of confirming this phenomenon. In this study, we examined the SLMD concentration-dependent effect on the radical scavenging activity of FA toward water-soluble ABTS^▪+^ radicals (Figure 5).

Figure 5a depicts the UV-visible absorption spectra after 2 h of reaction when ABTS radical solutions were added with various concentrations of SLMD in the presence of 0.048 mM FA. Half of the spectrum with a higher absorbance than 380 nm corresponds to the FA-free system (Figure 4a,b), and the region with a lower absorbance corresponds to the FA-added system. Furthermore, in the FA-added system, the absorbance of <370 nm was generally higher, whereas in the FA-free system, it was lower (Figure 5a,c). As reported earlier, ABTS^▪+^ radicals exhibit broad absorption around the maximum absorption at 732 nm and at approximately 420 nm, and these radicals are effectively scavenged by phenolic hydroxyl groups [27]. Conversely, when radicals are scavenged, ABTS radicals change into ABTS, causing an increase in absorption at 345 nm [27,33]. Therefore, our results indicate that FA addition scavenges ABTS^▪+^ radicals, resulting in the generation of the nonradical form ABTS. Moreover, no reduction in FA efficacy was observed with the addition of SLMD. Conversely, SLMD was confirmed to exhibit radical scavenging activity at higher concentrations than 4 mM by itself; it was also confirmed that SLMD exerts an additive effect with that of FA (Figure 5 and see Figure S2 in the Supplementary Materials). Furthermore, no radical scavenging activity of maltose (n = 2) was observed even at same weight concentration with 18 wt% (ca. 125 mM) SLMD, indicating the importance of amylose structure in the radical scavenging activity rather than total number of sugar moiety or non-reducing sugars, respectively. As shown in Figure 5d, there was a slight increase in radical scavenging activity with an increase in SLMD concentration in the FA-added system compared with that in the FA-free system. The difference between radical scavenging effects of SLMD–FA aq. and the sum of SLMD aq. and FA aq., respectively, showed a significant positive correlation with concentration or log(concentration) (see Figure S2 in the Supplementary Materials). It may be due to the interaction between SLMD and FA. Conversely, changes in solution viscosity due to increased SLMD concentration may have also influenced the results. For instance, the local concentration of FA or ABTS biradical may enhance the reaction efficiency. Further detailed and systematic investigations, including other carbohydrates, are required to draw conclusions.

### 3.5. Investigation of the Complexation Between SLMD and FA in Solid State

**Figure 6:**
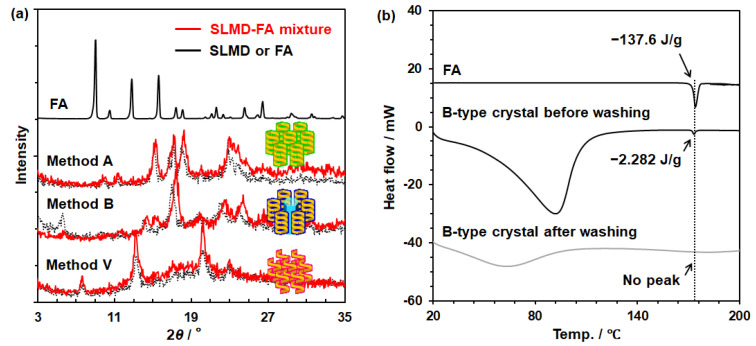
(**a**) PXRD profiles of FA and SLMD crystals prepared using Methods A, B, and V. (**b**) DSC thermograms of FA (3.8 mg) as well as FA-containing B-type SLMD crystals before (14.8 mg) and after washing with IPA (4.7 mg).

SLMD crystals obtained using Methods A, B, and V with or without the addition of FA were analyzed by powder X-ray diffraction (PXRD) (Figure 6a). In the PXRD profiles of SLMD crystals with and without FA addition, the formation of A-, B-, and V-type crystals was confirmed by the characteristic diffraction peaks [15]. Diffraction peaks at 15° and 23° for A-type crystals; 5.5°, 17.5°, 22°, and 24° for B-type crystals; and 7.5°, 13.5°, and 20.5° for V-type crystals, respectively, were confirmed. Nevertheless, only 1% of FA existed in A- and V-type SLMD crystals, whereas approximately 0.4 molar of FA compared with that of SLMD was recognized in B-type crystals, although the washing with IPA resulted in a complete loss of FA from the solid (Table 1). Based on these results, it was believed that SLMD in all crystals does not strongly interact with FA, whereas the B-type crystal appeared to possess a mechanism for containing FA during the solidification of SLMD.

Figure 6b displays the DSC thermogram for B-type SLMD containing FA before and after washing with IPA. For comparison, the DSC thermogram of FA is shown, and the melting temperature of FA was confirmed as 171 °C. Interestingly, a melting peak at 171 °C was detected in the DSC thermogram of FA-containing B-type SLMD before washing; however, the endothermic heat was only 1% of FA contained in the solid, demonstrating that most of FA existed without crystallization. Because FA slightly dissolves in water, it is possible that FA can be passively trapped with a large number of water molecules in the pore during the formation of B-type SLMD crystals (see Figure S3 in the Supplementary Materials) [15,34]. Since the pores of B-type crystals are large enough to trap numerous water molecules, it may be difficult to confirm the strong interaction; therefore, FA can easily be removed by washing with any solvent.

### 3.6. W/O Emulsion Systems

In Section 3.2, SLMD was found to enhance the solubility of FA in a water system. In addition, FA is known to provide additional UV protection [35]. In this section, we evaluated the UV protection ability of SLMD-FA mixtures in aqueous and W/O emulsion systems. The emulsion type was confirmed using a fluorescent microscope to examine curcumin-containing samples.

**Figure 7:**
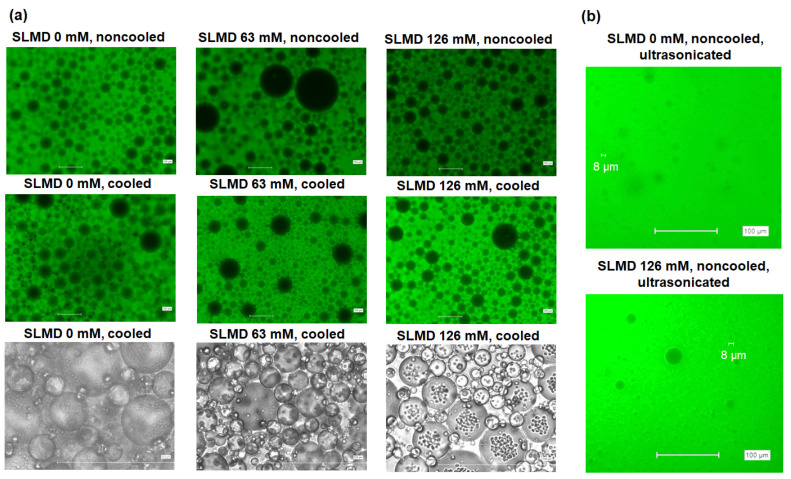
(**a**) Determination of emulsion type for the SLMD-aq.-TGO system at different SLMD concentrations using curcumin as oil indicator, and (**b**) determination of emulsion size for the emulsion used in the UV protection ability experiment. The top two photographs in (**a**) and the photographs in (**b**) show fluorescent images. The bottom photograph in (**a**) shows a phase-contrast microscope image. “Cooled” means the sample was stored at 4 °C overnight, and “noncooled” means the sample was stored without cooling, and “ultrasonicated” indicates that the sample was ultrasonicated after vortex mixing.

As shown in Figure 7, fluorescence was observed in the continuous layer of all samples, indicating that the oil layer containing fluorescent curcumin was on the outside and the water layer was on the inside. Notably, visible precipitation of SLMD crystals was observed when the emulsions were stored at 4 °C overnight, confirming a unique mode of crystallization within the emulsion (Figure 7a). The emulsion size prepared without ultrasonication was observed to be about several tens of μm in diameter, whereas the emulsion size prepared with ultrasonication was mostly smaller than 8 μm in diameter.

**Figure 8:**
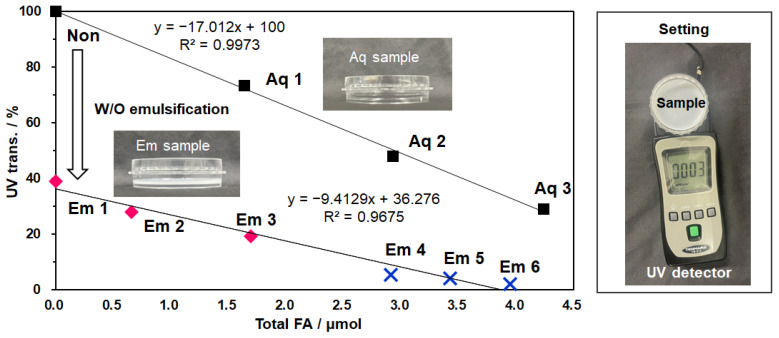
UV protection ability of SLMD-FA aq. and SLMD-FA aq.-TGO W/O emulsion as a function of total FA amount. Each aqueous solution (Aq) and emulsion sample (Em) is numbered as in Table S1, Supplementally Materials. The experimental setup is shown on the right.

As can be seen in Figure 8 and Table S1 in Supplementary Materials, UV transmittance of Aq samples (Aq 1–3) decreased linearly with increasing FA concentrations, a trend that was further enhanced by the addition of SLMD. Conversely, UV transmittance decreased for emulsion systems compared to a pure water system, even in the absence of FA. UV scattering was considered. In this system, the UV transmittance also decreased with FA concentration. It is noteworthy that the addition of SLMD to the aqueous system only (Em 1–3) and to both phases (Em 4–6) decreased linearly as a function of FA concentration. These results suggest that FA concentration is the primary determinant of UV transmittance in both systems and that the solubility enhancement provided by SLMD played a significant role.

Furthermore, the presence of SLMD was found to decrease the partition coefficient of FA from 2.44 to 0.96 as the SLMD concentration increased from 0 to 126 mM (Table 2). Since partition concentration (*P*) is defined as Log *P* = Log [FA in oil]/[FA in SLMD aq.] = Log [FA in oil] − Log [FA in SLMD aq.], an increase in FA solubility in SLMD aq. would be expected to decrease *p* values. Additionally, this suggests that adding SLMD allows more FA to transfer from the TGO layer to the water layer. Here, even though emulsifiers were absent, the transfer readily occurred with a vortex for 10 min owing to the formation of water soluble SLMD-FA complex.

Although CDs showed much higher *K*_1:1_ than SLMD, a large *K*_1:1_ is known to prevent FA from permeating the skin layer [36]. SLMD is expected to enhance FA concentration in the aqueous system without disturbing FA permeation into the skin layer. FA has been reported as an effective anti-aging component, so further research into SLMD-FA mixture formulations for skincare products would be interesting. Alternatively, an O/W emulsion using SLMD aqueous solution and plant oil has been reported [16], and this study confirmed the stabilization of a W/O emulsion using SLMD aqueous solution and 5% emulsifier in hexadecane in terms of suppressing oil floating (see Figure S4 in the Supplementary Materials). Further investigation into SLMD in relation to surfactants and lipids would be significant for the development of adequate formulations. On the other hand, the specific gravity of TGO is 0.95, which is close to the value of 1.0 for water. However, the specific gravity of hexadecane is 0.776, which is far from 1.0. Therefore, TGO was assumed to be a more adequate oil that prevents oil from floating due to the difference in specific gravity between the water and oil phases (see Figure S4 in the Supplementary Materials).

## 4. Conclusions

In this experimental investigation of the molecular interaction between SLMD and FA in water, SLMD and FA exhibited a 1:1 noncovalent interaction, and the change in the ellipticity of circular dichroism and the multipoint interaction between SLMD and FA strongly suggested that a complex is formed by encapsulating FA within the helical structure of SLMD. This finding is consistent with previous DFT calculation results that showed the inclusion formation between amylose and FA [17], thereby strongly supporting the prediction. Our results demonstrate that SLMD with n = 9.7 can form complexes with components other than iodine and also suggest that SLMD is useful for discussing the interaction between amylose structure and other components. Moreover, our findings suggested that FA was passively trapped in the B-type SLMD crystal. Hence, developing materials that incorporate other components into the crystalline structures can be effective for applications in food, cosmetics, and pharmaceuticals. Furthermore, the usefulness of SLMD in enhancing UV protection was confirmed in both aqueous and W/O emulsion systems. In the near future, we would like to verify the effectiveness of skin administration.

## Figures and Tables

**Table 1:** FA/SLMD molar ratio in FA–SLMD B-type crystal before and after washing with solvent.

	B-Type Crystal			
Used Solvent	Before	Washed with Acetone	Washed with EtOH	Washed with IPA
FA/SLMD (mol/mol)	0.43 *	0.00	0.00	0.00

* For this calculation, the purity of B-type crystal was estimated as 60% based on the result of Figure S3, Supplementary Materials.

**Table 2:** The partition coefficients [FA in TGO]/[FA in SLMD aq.] with different SLMD concentrations.

0 mM	63 mM	126 mM
2.43	1.44	0.96

## Data Availability

Data is contained within the article or Supplementary Material.

## Supplementary Materials

The following supporting information can be downloaded at https://www.mdpi.com/article/10.3390/polym18020166/s1, Figure S1: ^1^H-NMR analysis of CD-FA aq. solution; Figure S2: The difference in absorbance between the SLMD-FA aqueous solution and the sum of the SLMD aqueous solution and the FA aqueous solution; Figure S3: TG charts of A-, B-, and V-type SLMD crystals and geometrical illustrations of SLMD–FA mixture; Figure S4: Effect of SLMD on emulsion stability. Table S1: Samples of SLMD aq. and W/O emulsion.

## Abbreviations

The following abbreviations are used in this manuscript:

SLMD	Short linear maltodextrin
FA	Ferulic acid
UV	ultraviolet
ABTS	2,2′-Azinobis(3-ethylbenzothiazoline-6-sulfonic Acid Ammonium Salt)
CD	Cyclodextrin
TGO	Triethylhexanoin
NMR	Nuclear magnetic resonance
ROESY	Rotating-frame nuclear Overhauser effect spectroscopy
TG	Thermogravimetric
DSC	Differential scanning calorimetry
PXRD	Powder X-ray diffraction
