# Supplementary material for: Molecular Association Between Short Linear Maltodextrin and Ferulic Acid and the Exploration of Its Applicability
Source: Polymers (Basel). 2026 Jan 7;18(2):166. doi: 10.3390/polym18020166 (PMC12846177; doi:10.3390/polym18020166)
Supplement: Supplementary file 1 [file polymers-18-00166-s001.zip › polymers-4058129-supplementary.pdf]

Supplementary Materials

Molecular Association between Short Linear Maltodextrin and Ferulic Acid and the Exploration of its Applicability

Shigesaburo Ogawa[1,*], Daisuke Sugitani[1], Minenosuke Matsutani[1], Mizuho Takayashiki[2], Atsushi Kawano[2]

[1] Department of Food, Aroma and Cosmetic Chemistry, Faculty of Bio-industry, Tokyo University of Agriculture, 196, Yasaka, Hokkaido 099-2493, Japan; so207914@nodai.ac.jp
[2] Research & Engineering Department, Showa Sangyo Co., Ltd., 2—20 —2 Hinode, Funabashi-shi, Chiba 273—0015, Japan; atsushi_kawano@showa-sangyo.jp
* Correspondence: so207914@nodai.ac.jp

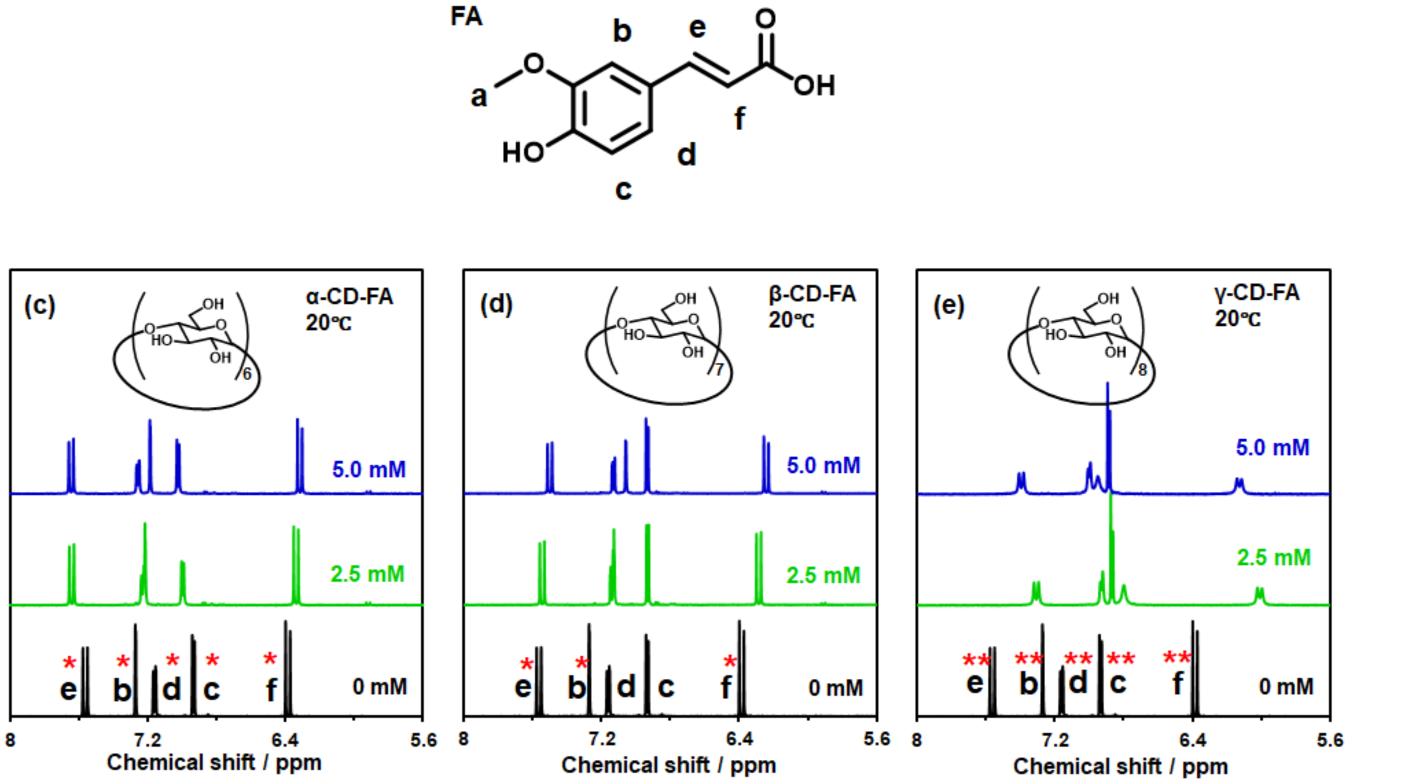

Figure S1. Comparison of ^{1}H-nuclear magnetic resonance (NMR) spectra for various cyclodextrin (CD)-saturated ferulic acid (FA) aq. solutions at different CD concentrations (20°C, D_2O). The asterisk indicates that the signal exhibits concentration dependence.

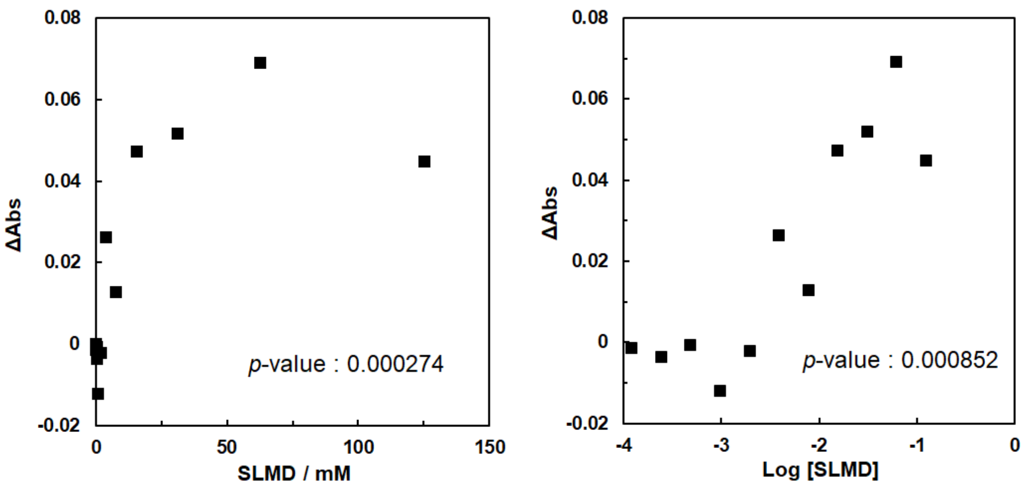

Figure S2. The differences in absorbance between SLMD-FA aq. and the sum of SLMD aq. and FA aq. are shown as a function of (a) SLMD concentration in mM and (b) log [SLMD in M]. Regression analysis revealed a significant deviation from additivity, indicating that the antioxidant effect of the combined system cannot be explained by a simple additive model.

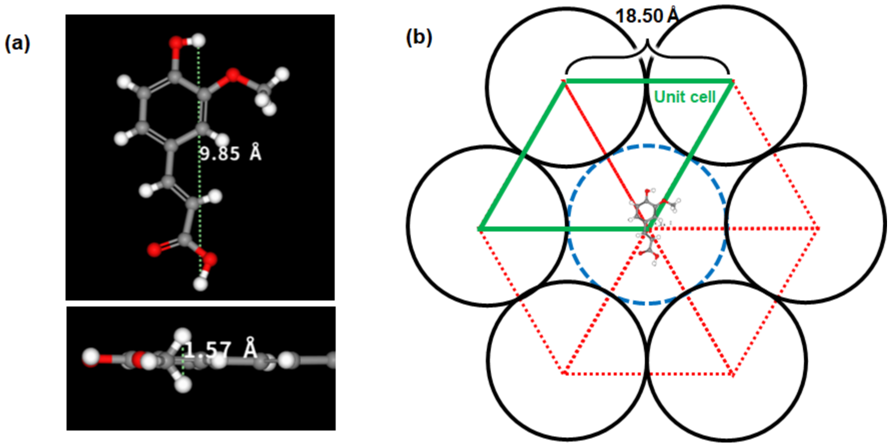

Figure S3. Geometrical illustrations of (a) FA and (b) SLMD–FA mixture, where FA can exist in the pore of B-type SLMD crystals.

In B-type crystals, there were large pores in the center area, and a large amount of water filled [15]. Fig. S3a illustrates the molecular length of FA, with 9.85 Å and 1.57 Å being the maximum and minimum lengths. Fig. S3b shows the geometrical images from the top of B-type SLMD containing FA in the large pore inside of the six double helices of SLMD. Since the diffraction patterns were similar to those of starch molecules, it was assumed that the crystalline domains with large pores at the centre of a six-double-helix structure were present. In this case, apparently, the pore size was so large that FA molecules could be trapped inside the pore of B-type-SLMD crystals. Conversely, from the side aspect, because the elongated molecular length of the double helix of 10 glucose units was 33.3 Å [34], 21 FA molecules of length 1.57 Å can be contained. In that case, the molar ratio of FA with SLMD would reach about 5.25 for unit cell contains four per three SLMD molecules and twenty-one per three FA molecules; however, the experimental result revealed 0.43 (Table 1).

Table S1. Samples of SLMD aq. and W/O emulsion prepared with SLMD aq.-TGO, total FA amount and UV transmittance % (UV-trans). The initial [FA] refers to the FA concentration in the solution used to prepare the emulsions. Total FA values represent the total amount of FA added, and these values were not estimated after emulsion preparation.

Sample	SLMD	Initial [FA] in W	Initial [FA] in O	Vol of W	Vol of O	Total FA	UV trans.
	mM	mM	mM	mL	mL	μmol	%
**Non**	0	0	No oil phase	0.40	0	0.00	100.0
**Aq 1**	0	4.1	No oil phase	0.40	0	1.6	73.2
**Aq 2**	63	7.3	No oil phase	0.40	0	2.9	47.9
**Aq 3**	126	10.6	No oil phase	0.40	0	4.2	29.0
**Em 1**	0	0	0	0.16	0.24	0	39.0
**Em 2**	0	4.1	0	0.16	0.24	0.7	27.8
**Em 3**	126	10.6	0	0.17	0.23	1.9	19.2
**Em 4**	0	4.1	9.4	0.16	0.24	2.9	5.2
**Em 5**	63	7.3	9.4	0.17	0.23	3.4	4.0
**Em 6**	126	10.6	9.4	0.17	0.23	4.0	2.1

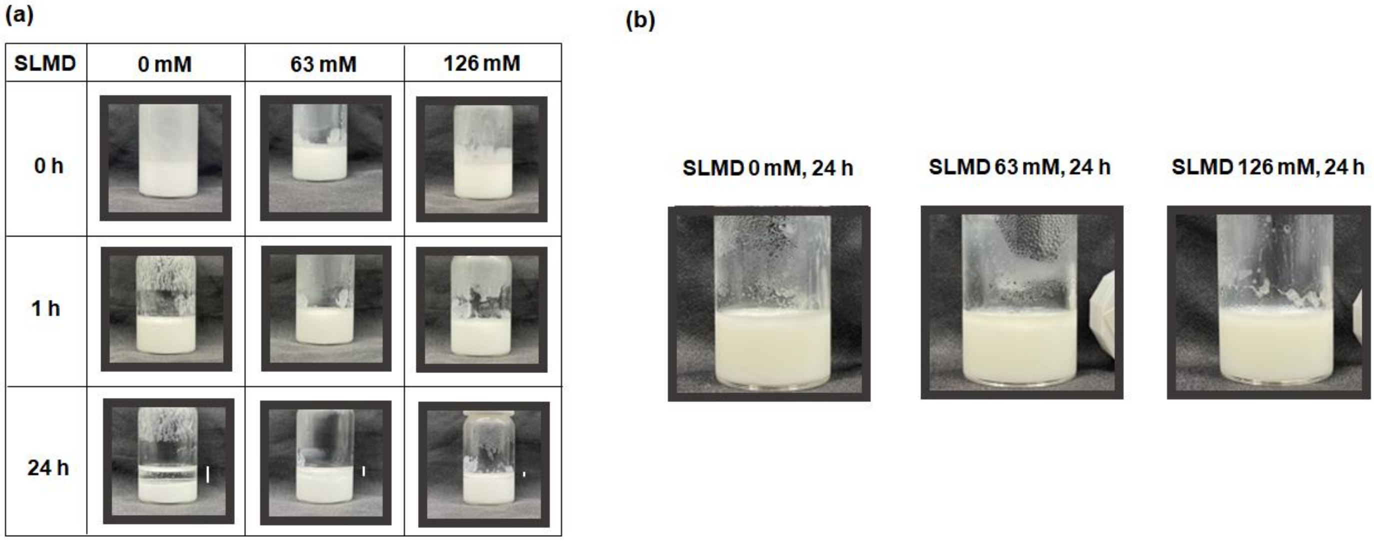

Figure S4. Effect of SLMD on emulsion stability: (Left) W/O emulsion using hexadecane; (right) TGO as the oil phase. The hexadecane system showed significant destabilization after 24 hours' storage in the absence of SLMD. The TGO system was more stable and was therefore used for the overall study.
